# Is there agreement across diagnostic instruments in the identification of the impairment in intrinsic capacity later in life? A cross-sectional study with community-dwelling older adults

**DOI:** 10.1016/j.jnha.2026.100829

**Published:** 2026-04-01

**Authors:** Luana A. Soares, Leonardo A.C. Teixeira, Juliana N.P. Nobre, Maria Fernanda S. Mourão, Maria Clara M. Oliveira, Bárbara R. Barbosa, Ana Flávia V. Trindade, Gabriele T. Gonçalves, Jousielle M. Santos, Joyce N. Santos, Henrique S. Costa, Pedro H.S. Figueiredo, Murilo X. Oliveira, Ronaldo L. Thomasini, Núbia C.P. Avelar, Redha Taiar, Lucimere Bohn, Adérito Seixas, Vanessa A. Mendonça, Ana Cristina R. Lacerda

**Affiliations:** a Universidade Federal dos Vales do Jequitinhonha e Mucuri, Diamantina, Minas Gerais, Brazil; b Universidade Federal da Bahia, Bahia, Brazil; c Universidade Federal de Santa Catarina, Santa Catarina, Brazil; d Université de Reims Champagne, Ardenne, Reims, France; e Research Center in Physical Activity, Health and Leisure, Faculty of Sports, University of Porto, and Laboratory for Integrative and Translational Research in Population Health, Porto, Portugal; f Universidade Fernando Pessoa, Porto, Portugal

**Keywords:** Healthy aging, WHO, Prevalence, Aging.

## Abstract

**Objectives:**

To compare and investigate agreement across diagnostic instruments in detecting impairment in the domains of intrinsic capacity (IC).

**Design:**

Cross-sectional descriptive design. Setting and participants: The study was conducted with 205 community-dwelling older adults (ages 60–96; mean: 71.3 ± 7.9 years).

**Measurements:**

IC was assessed across five domains, using multiple diagnostic instruments for each domain: cognition [Mini-Mental State Examination (education-adjusted versus raw score of 26 points) and a cognitive battery]; mobility (Short Physical Performance Battery, gait speed test, chair stand test, and Timed Up and Go test); vitality (Mini Nutritional Assessment, handgrip strength, body mass index, and self-reported unintentional weight loss); psychological well-being (Geriatric Depression Scale, Center for Epidemiologic Studies Depression Scale, and self-reported of mental health diagnosis or use of antidepressant or anxiolytic medications); and sensory function (whisper voice test, Snellen chart, and self-reported hearing or visual impairment). McNemar’s test and Cochran’s Q-test were used to compare the detection of impairment in IC domains. Cohen’s Kappa and Fleiss’ Kappa tests were used to measure the level of agreement across diagnostic instruments.

**Results:**

Detection of impairment in each IC domain varied substantially across diagnostic instruments. Moderate agreement was found in the diagnostic instruments for mobility (K = 0.64) and hearing (K = 0.69; agreement = 88.8%).

**Conclusions:**

Diagnostic instruments substantially influence the detection of impairment on IC. Moderate agreement was found for mobility and hearing measures, underscoring the urgent need for harmonized assessment protocols and highlighting the critical impact of instrument selection on estimating the prevalence of impairment on IC.

## Highlights

• Diagnostic instruments substantially impact the detection of impairment on IC domains.
• There is moderate agreement across diagnostic instruments in the mobility domain.
• There is moderate agreement across diagnostic instruments in the hearing domain.
• There is a lack of agreement across diagnostic instruments in some domains of IC.

## 1 Introduction

Intrinsic capacity (IC) is defined as the aggregate of an individual’s physical and mental capacities across the course of life [1,2]. It reflects one’s overall functional abilities in terms of cognition, mobility, vitality, psychological well-being, and sensory function [1,2]. In 2025, the World Health Organization (WHO) updated the Integrated Care for Older People (ICOPE) guidelines to operationalize the screening and management of IC along the aging process [2].

The ICOPE framework consists of two main assessment steps. In the first step, initial screening is performed of each of the five IC domains using a simple tool. Individuals with possible decreased IC should then undergo an in-depth assessment to confirm the presence of impairment (step 2) [2]. However, evidence on the performance of the screening tool is scarce [3], and measures for different domains and cutoff points have not yet been standardized [4]. Heterogeneity among instruments may influence prevalence estimates of impairment on IC. To date, no study has performed a head-to-head comparison of multiple diagnostic instruments applied to the same individuals across all five IC domains. Evidence from studies on other health conditions related to aging has shown that the choice of diagnostic instrument can significantly influence prevalence estimates [5,6].

As global interest in healthy aging continues to increase, a more comprehensive understanding of the prevalence of impairment on IC could contribute to advancing the discussion on preserving functional capacity in older populations. Preliminary data from a scoping review (in progress) and previous systematic reviews [[7], [8], [9]] identified the instruments most commonly used to assess each of the IC domain. Building on this evidence, the present study aimed to compare and investigate the level of agreement among diagnostic instruments used to detect impairment in IC domains.

## 2 Methods

### 2.1 Study design and participants

A cross-sectional study was conducted with community-dwelling older adults residing in urban areas of the municipality of Diamantina (Minas Gerais, Brazil) from March 2023 to March 2024. The sample was selected by drawing from the twelve primary care units in the municipality. The selected primary care units included a total of 6,139 older adults with complete registration details and contact information. Individuals were selected for random sampling weighted by primary care unit. Individuals were recruited through phone calls or face-to-face invitations at their homes.

Community-dwelling older adults (60 years of age or older) were recruited, irrespective of sex or race. The exclusion criteria were (i) score of 7, 8, or 9 on the Clinical Frailty Scale [10] and (ii) failure to complete the tests. Following the application of the inclusion and exclusion criteria, eligible volunteers were assessed at the Universidade Federal dos Vales do Jequitinhonha e Mucuri (UFVJM). All participants signed a written consent form.

The sample size was calculated considering both prevalence estimation and agreement analysis. Initially, the sample size was estimated using OpenEpi based on a population of 6,139 community-dwelling older adults and a 25.2% prevalence of IC decline [11], assuming a 5% absolute precision and a 90% confidence level, resulting in a minimum of 205 participants. Additionally, an agreement-based appraisal was performed using the hypothesis-testing framework for Cohen’s kappa proposed by Donner and Eliasziw. Assuming a dichotomous outcome, an expected prevalence of 25%, α = 0.05 (two-sided), 80% power, and a conservative contrast between κ₀ = 0.40 and κ₁ = 0.60, the required sample size was approximately 178 participants. These κ parameters were defined based on previous cross-sectional studies comparing diagnostic instruments in older adults that reported poor-to-moderate agreement [6]. As the final analytical sample included 205 participants, the study was adequately powered for both prevalence estimation and agreement analysis.

This study received approval from the UFVJM Institutional Review Board (protocol no. 5.862.740). The procedures were applied in accordance with the principles outlined in the Declaration of Helsinki [12]. The study was reported following the Strengthening the Reporting of Observational Studies in Epidemiology guidelines [13].

### 2.2 Instruments

2.2.1. Sociodemographic and clinical characteristics: Participants reported about medication use, living condition (whether they lived alone or with family), and years of schooling. Frailty status was assessed using the Clinical Frailty Scale, which ranges from 1 (very fit) to 9 (terminally ill) [10]. Physical activity level was estimated using the International Physical Activity Questionnaire (short form), classifying individuals as very active, active, irregularly active, or sedentary [14].

2.2.2. Intrinsic capacity: IC was assessed using the five components recommended by the WHO for an in-depth assessment [2]. Multiple diagnostic instruments were used for each domain:

The cognitive domain was assessed using the Mini-Mental State Examination (MMSE) [15], applying both education-adjusted cutoff points [16] and a fixed cutoff score of 26 points [7,17], as well as a brief cognitive battery assessing temporal and spatial orientation and recall [18]. Cognitive decline was defined as scores below the MMSE cutoff or errors in orientation or recall on the cognitive battery.

The locomotion domain was assessed using the Short Physical Performance Battery (SPPB), usual gait speed, the five-times chair stand test, and the Timed Up and Go (TUG) test. Limited locomotion was defined as SPPB ≤ 9 points [2,19], usual gait speed ≤0.8 m/s [20,21], five-times chair stand test >15 seconds or inability to perform the test [22], or TUG ≥ 20 seconds [23].

The vitality domain was assessed using the Mini Nutritional Assessment (MNA), handgrip strength (HGS), body mass index (BMI), and self-reported unintentional weight loss. Malnutrition (Vitality impairment) was defined as MNA ≤ 23.5 points [24], HGS < 27 kgf for men and <16 kgf for women [25,26], BMI < 22 kg/m² [27,28], or self-reported unintentional weight loss.

The psychological domain was assessed using the 15-item Geriatric Depression Scale (GDS-15), the Center for Epidemiologic Studies Depression Scale (CES-D), and self-report of clinical diagnosis of mental health problem or use of antidepressant or anxiolytic medication. Depressive symptoms (Psychological impairment) was defined as GDS-15 > 5 points [29,30], CES-D ≥11 points [31], or self-reported of diagnosis of mental health disorder or use of antidepressant or anxiolytic medication.

The sensory domain was assessed through hearing and visual evaluations using the whisper voice test, the Snellen chart, and self-reported hearing and/or visual impairment. Sensory impairment was defined as hearing loss (fewer than 3 words correctly repeated during the whisper voice test) [2], visual acuity worse than 20/25 on the Snellen chart [32,33], or self-reported hearing problems, deafness, or visual impairment.

Additional details regarding the definition, measurement procedures, and cutoff criteria for each intrinsic capacity domain are provided in Supplementary Table S1.

### 2.3 Statistical analysis

Data were analyzed using Statistical Package for the Social Sciences, version 25.0 (SPSS Statistics; IBM, Armonk, NY, USA). Continuous variables were expressed as mean and standard deviation, while categorical variables were expressed as frequency and percentage. The detection rates of the impairment on IC domains were described as relative frequency (%).

Comparisons of the detection of impairment in IC domains across the different diagnostic instruments were performed using either McNemar’s test or Cochran’s Q-test. Cohen’s Kappa test and Fleiss’ Kappa test were used to measure the level of agreement among diagnostic tools for impairment on IC. The classification categories proposed by McHugh (2012) [34] were considered in the interpretation of the level of agreement: 0 to 0.20 represents no agreement; 0.21 to 0.39 represents minimal agreement; 0.40 to 0.59 represents weak agreement; 0.60 to 0.79 represents moderate agreement; 0.80 to 0.90 represents strong agreement; and above 0.90 represents nearly perfect agreement. The significance level adopted for the analyses was 5%.

## 3 Results

Six hundred twenty-seven community-dwelling older adults were initially recruited for participation. After applying the exclusion criteria, 205 were enrolled in the study (age range: 60–96 years; mean: 71.3 ± 7.9 years). Most participants were women (n = 135; 65.9%). A total of 87.3% (n = 179) lived with their families, 55.1% (n = 113) had less than eight years of schooling, and 8.8% (n = 18) were illiterate. The participants took an average of 3.8 (± 2.7) medications. Regarding frailty, most were classified as having mild frailty (n = 76, 37.1%) and were managing well (n = 57, 27.8%) (scores 4 and 3 on the Clinical Frailty Scale, respectively). In terms of physical activity level, 8.8% (n = 18.0) and 66.3% (n = 136) were classified as very active and active, whereas 6.8% (n = 14) were classified as sedentary.

**Fig. 1:**
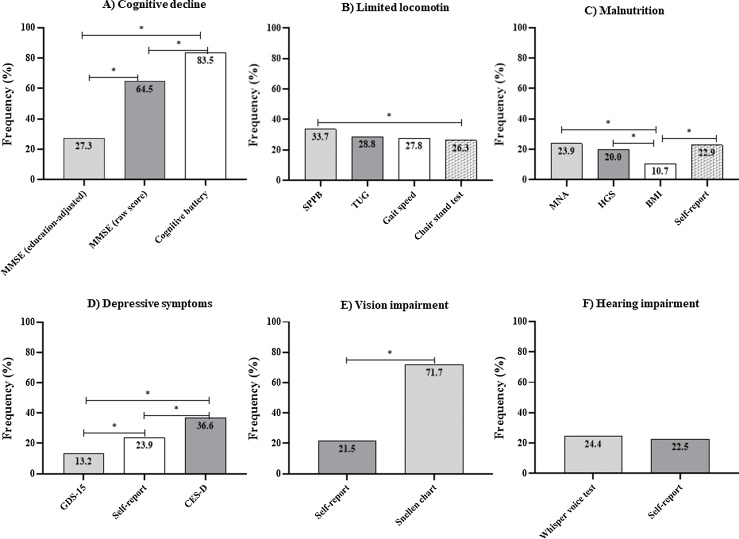
Detection of impairment in IC domains (%) across diagnostic instruments. Difference in detection of impairment in IC domains in community-dwelling older adults across diagnostic instruments. a) Cognitive domain: b) Mobility domain: c) Vitality domain: d) Psychological domain; e) Sensory domain (visual acuity); f) Sensory domain (hearing acuity). Note: BMI: Body Mass Index; GDS: Geriatric Depression Scale; HGS: Handgrip Strength; MNA: Mini Nutritional Assessment; MMSE: Mini-Mental State Examination; SPPB: Short Physical Performance Battery; TUG: Timed Up and Go test. *Statistical significance (p < 0.05).

The detection of impairment in IC domains varied substantially depending on the diagnostic instrument used (Fig. 1).

Cognitive domain: The cognitive battery detected the highest frequency of cognitive impairment (83.4%), followed by the MMSE (raw score of 26 points) (64.4%). The MMSE adjusted for educational level identified a 27.3% frequency of cognitive impairment. Cochran’s Q test revealed that the difference between proportions was statistically significant when comparing the three diagnostic instruments (χ^2^(2) = 145.54, p < 0.001) (Fig.1a). Fleiss’ Kappa test revealed a lack of agreement among the three diagnostic instruments (K = 0.056; 95%CI = −0.023–0.135; p = 0.16), and the confidence interval also indicates limited precision and instability of the agreement estimate.

Mobility domain: The limited locomotion was 33.7%, 28.8%, 27.8%, and 26.3% when using the SPPB, TUG test, gait speed test, and chair stand test, respectively. A statistically significant difference in mobility impairment was found among the four diagnostic instruments (χ^2^(3) = 8.40, p = 0.03). Pairwise comparisons revealed that the frequency of mobility impairment was significantly higher when using the SPPB compared to the chair stand test (Fig. 1b). Fleiss’ Kappa test indicated moderate agreement among the four diagnostic instruments (K = 0.644; 95%CI = 0.588–0.700; p < 0.001), with a narrow confidence interval, reflecting high stability and precision of the agreement estimate.

Vitality domain: The MNA was the instrument that identified the highest number of cases of vitality impairment (23.9%), followed by the self-report question (22.9%), HGS (20.0%), and BMI (10.7%). Cochran’s Q test revealed a statistically significant difference in vitality impairment among these instruments (χ^2^(3) = 18.18, p < 0.001). Pairwise comparisons revealed that the difference was between BMI and the other instruments (Fig. 1c). Fleiss’ Kappa test indicated a lack of agreement among the four diagnostic instruments (K = 0.15; 95%CI = 0.094–0.205; p < 0.001). The relatively narrow confidence interval suggests acceptable stability and precision despite the low magnitude of agreement.

Psychological domain: Depressive symptoms were identified in 36.6% of participants using the CES-D, 23.9% with the self-report of a clinical diagnosis of a mental health problem, and 13.2% with the GDS-15. Cochran’s Q test revealed a statistically significant difference in the frequency of depressive symptoms among the three diagnostic instruments (χ^2^(2) = 36.08; p < 0.001) (Fig. 1d). Fleiss’ Kappa test showed a lack of agreement among the three diagnostic instruments (K = 0.156, 95%CI = 0.077–0.235; p < 0.001), with the confidence interval indicating moderate precision of the estimate.

Sensory domain: The self-report questions identified the lower rates of sensory impairment. Visual impairment was identified in 71.7% and 21.5% of participants when assessed using the Snellen chart and the self-report question, respectively. McNemar’s exact test showed a statistically significant difference in the visual impairment between the two diagnostic instruments (χ²(1) = 78.226; p < 0.001) (Fig. 1e). Cohen’s Kappa test showed no agreement between the two diagnostic instruments (K = −0.040; 95%CI = −0.123–0.044; p = 0.335; 35.1% agreement), with the confidence interval indicating limited precision and instability of the agreement estimate.

The detection of hearing impairment were 22.9% and 24.4% when using the self-report question and whisper test, respectively. McNemar’s exact test showed no statistically significant difference in the hearing impairment between the two diagnostic instruments (χ^2^(1) = 0.174; p = 0.678) (Fig.1f). Cohen’s Kappa test revealed moderate agreement between the two diagnostic instruments (K = 0.689; 95%CI = 0.552–0.800; p < 0.001; 88.8% agreement), with a narrow confidence interval, reflecting stability and precision of the agreement estimate.

Pairwise comparisons of the Kappa analysis results between groups are displayed in Supplementary Table S2.

## 4 Discussion

This study provides one of the first within-head-to-head comparisons of multiple diagnostic instruments used to operationalize IC domains in community-dwelling older adults.The main findings revealed that (1) the choice of diagnostic instrument influences the detection of impairment in each IC domain; (2) the level of agreement among instruments used to operationalize the domains is low - only the instruments employed for the mobility and sensory (hearing) domains achieved moderate agreement. These results are clinically relevant, as such instruments are used in the in-depth assessment phase to confirm impaired IC (step 2), which is used for the development of a personalized care plan to reverse or slow these declines [2]. I.e., an individual's IC profile may differ markedly depending on the tools used in a screening setting, which could affect care pathways. Therefore, the discrepancies have direct clinical relevance, potentially altering both the identification of cases and downstream management strategies.

Our findings are consistent with data from systematic reviews, which have shown a variation in the prevalence of impairment on IC due to the heterogeneity of diagnostic instruments [[7], [8], [9],^35^]. This heterogeneity underscores the need for the standardization of assessment protocols and cutoff points to enhance the comparability and clinical applicability of the IC framework proposed by the WHO. We hypothesize that the low level of agreement among diagnostic instruments observed in our study reflects the inherent variability, which may be attributed to differences in the diagnostic accuracy (sensitivity and specificity) of each instrument.

Cognitive domain: Most standard assessments of cognition require a minimal level of schooling [2], which may introduce bias when applied to populations with low literacy [16,36]. Thus, education-adjusted cutoff points are recommended to minimize the occurrence of misclassifications [36]. Previous studies have demonstrated that the education-adjusted MMSE has high specificity, while the raw MMSE score has high sensitivity [7,16,37]. According to the ICOPE/WHO framework, when standardized cognitive instruments are not available, self-reports on memory, orientation, speech, and language problems can be used as indicators of cognitive decline [2]. González-Bautista et al. (2025) [38] reported that the cognitive test battery has high sensitivity and low specificity values. In our study, the cognitive battery identified the highest proportion of cognitive decline, probably due to its lower specificity. To date, no study has compared the diagnostic accuracy of the cognitive test battery to standardized instruments.

Mobility domain: The instruments for assessing locomotor capacity achieved a moderate level of agreement. The SPPB is the test recommended by ICOPE/WHO for measuring locomotor capacity [2]. However, its subcomponents, namely the gait speed test and the chair stand test, are also reported [7]. In our study, the pairwise comparisons showed a difference in the proportion of locomotion impairment detection only between the SPPB and the chair stand test. According to Ortiz et al. (2021) [7], the SPPB has high specificity, while the chair stand test has high sensitivity. For this reason, ICOPE/WHO recommends using both tests for screening and in-depth assessments, as the sensitivity and specificity values complement each other. There was no difference in the detection of impairment in locomotion between the SPPB, TUG test, and gait speed test. This similarity is supported by the strong correlation among the tests [39]. Sutil et al. (2023) [6] also demonstrated moderate agreement among the three diagnostic tests for detecting severe sarcopenia (using HGS and appendicular lean mass relative to height). In our study, we used the gait speed test and TUG test cutoff points proposed by the European Working Group on Sarcopenia (2019) [22], as specific cutoff points for detecting limited locomotion within the IC construct are currently unknown.

Vitality domain: The MNA, HGS, and self-reported weight loss revealed similar rates detecting of impairment in vitality. The MNA has good diagnostic accuracy (sensitivity: 90%; specificity: 87%) [7] as well as a strong correlation with HGS and self-reported weight loss [40], which may explain the similar detection rates among the three diagnostic tests. To our knowledge, the HGS cutoff points for the screening of the risk of undernutrition among Brazilian older adults have not yet been investigated. Therefore, we used the cutoff points recommended by the European Working Group on Sarcopenia (2019) [22]. Our results demonstrated a significant difference between the MNA and BMI in detecting the impairment in vitality. This finding is consistent with data described by Pereira et al. (2021) [41], who similarly reported substantial discrepancies in the prevalence of nutritional risk when comparing the MNA and BMI, indicating that these measures do not consistently classify the same individuals. Although anthropometric indicators are included in the MNA, the use of BMI alone, particularly with the cutoff of <22 kg/m^2^ [27,28], has not yet been systematically validated as an alternative to the MNA in Brazilian older adults. Additionally, evidence from Ethiopia suggests that an even lower BMI threshold (<18.5 kg/m²) may be required for the accurate identification of malnutrition in older adults [42].

Psychological domain: The detection rate of depressive symptoms was higher when using the CES-D compared to both a clinical diagnosis and the GDS-15. These results are compatible with findings from previous validation studies, which indicated that the CES-D cutoff has a low positive predictive value [43], suggesting that this instrument may overestimate the proportion of individuals at risk for depression [31]. Differences in item content may partially explain this inflation in prevalence: the CES-D includes somatic symptoms, such as sleep disturbances, fatigue, and changes in appetite, which are highly prevalent among older adults due to multimorbidity and chronic conditions. As a result, older adults completing the CES-D may report somatic complaints related to physical illness rather than depressive psychopathology, leading to higher classification rates compared to the GDS-15. Regarding the clinical diagnosis, our findings are compatible with those described by Sjöberg et al. (2017) [44], who investigated the prevalence of depression in older adults in Sweden using different diagnostic criteria. Only one-third of older adults with clinically identified depression were receiving antidepressant treatment, suggesting that underdiagnosis and underreporting are common in this population.

Sensory domain: Our findings revealed a discrepancy in visual impairment when self-reported and measured using the Snellen chart. Previous studies have shown that self-report measures achieve low agreement with objective assessments [45] and have low sensitivity [46], which can result in a substantial underestimation of visual impairment. These limitations are partially explained by the fact that many older adults adapt to the gradual loss of vision or attribute the symptoms to the normal aging process, resulting in incorrect classifications [45]. In contrast, the Snellen chart (<20/30) has high sensitivity when compared to an ophthalmological examination for detecting any ocular disease in the worse eye [47]. Detection of hearing impairment was similar between the whisper test and the self-assessment questionnaire, and the instruments demonstrated moderate agreement. A systematic review showed that these are reliable screening tests for age-related hearing loss and have sensitivity and specificity higher than 70% for adults ≥65 years of age compared to pure-tone audiometry [48].

It is important to note that the stability of kappa statistics varied across domains. Mobility and hearing demonstrated the most precise and stable estimates, while vitality and psychological domains showed weak agreement, though statistically consistent, with moderate precision. In contrast, cognitive and vision showed unstable (confidence intervals crossing zero), suggesting limited precision and uncertainty regarding the presence of agreement. Therefore, the interpretation of the agreement estimates in this study prioritized not only the point estimates but also the range and limits of the respective 95% confidence intervals. In domains where the confidence intervals were wider or crossed zero, the results should be interpreted with caution.

The findings of this study suggest that these issues must be considered in the discussion on the concept and assessment of IC, which could help identify patients with decline in the domains of this construct. The findings also underscore the need for the standardization of instruments and cutoff points. Future studies should compare these diagnostic instruments with the gold standard. Additionally, it is necessary to validate specific IC decline cutoff points for certain instruments (TUG test, gait speed test, HGS, BMI, and CES-D), validate self-report measures, and directly compare ICOPE Stage 1 screening tools to establish standardized diagnostic instruments. This study has several strengths that should be highlighted. First, we used the most commonly reported diagnostic instruments to assess all IC domains. Second, we used validated instruments, versions, and cutoff points adapted for Brazilian older adults, when available, and cutoff points recommended by the ICOPE/WHO. Third, the recruitment of older adults from diverse locations enabled us to obtain data that more accurately reflect the diversity of community-dwelling older adults, thus contributing to ecological validity. However, due to the marked socioeconomic and geographic heterogeneity in the Southeast region of Brazil, the findings should be interpreted with caution and cannot be extrapolated to all older adults in the country. In particular, the relatively low educational level may have influenced performance on cognitive instruments, many of which are known to be sensitive to schooling. Therefore, agreement patterns observed in the cognitive domain may differ in populations with higher educational attainment. Replication in multicenter and nationally representative studies is necessary to confirm the stability of these findings across diverse contexts. Important limitations must also be acknowledged. The absence of gold-standard instruments (e.g., audiometry for hearing loss, comprehensive neuropsychological testing for cognition, or ophthalmologic examination for vision) constitutes a limitation of our study, impeding definitive conclusions about each instrument and inferences about the best choice. As a result, we were not able to evaluate sensitivity, specificity, or overall diagnostic accuracy. Therefore, because no gold-standard reference assessments were included, our findings should be interpreted strictly as evidence of inter-instrument variability rather than diagnostic accuracy. To overcome these limitations, future studies should incorporate criterion-standard assessments to determine the sensitivity, specificity, and predictive value of commonly used IC instruments. In addition, further research is needed to determine whether there are also differences in the potential predictors across IC domains when using different diagnostic instruments.

## 5 Conclusion

The selection of diagnostic instruments influences the detection of impairment in the domains of IC. Moderate agreement was found across diagnostic instruments only in the mobility and sensory (hearing) domains. The present findings suggest that these issues must be considered in the discussion on the concept and assessment of IC.

## CRediT authorship contribution statement

Conceptualization: LAS, LACT, JNPN, MFSM, MCMO, BRB, AFVT, GTG, JMS, JNS, HSC, PHSF, MXO, RLT, NCPA, RT, LB, AS, VAM, ACRL. Acquisition of data: LAS, MFSM, MCMO, BRB, AFVT. Analysis and interpretation of data: LAS, MXO, NCPA, LB, ACRL. Writing – original draft, Writing – review & editing: LAS, LACT, JNPN, MFSM, MCMO, BRB, AFVT, GTG, JMS, JNS, HSC, PHSF, MXO, RLT, NCPA, RT, LB, AS, VAM, ACRL. Supervision: ACRL.

## Declaration of Generative AI and AI-assisted technologies in the writing process

Authors did not use generative artificial intelligence (AI) and AI-assisted technologies in the writing process.

## Funding statement

This work was supported by the following Brazilian fostering agencies: *Fundação de Amparo à Pesquisa do Estado de Minas Gerais* (FAPEMIG) (2070.01.0005826/2021-36; APQ-02018-24; APQ-04955-23; AQP-00797-26), *Conselho Nacional de Desenvolvimento Científico e Tecnológico Universal* (CNPq) (303688/2024-6; 303706/2024-4), and *Coordenação de Aperfeiçoamento de Nível Superior* (CAPES) - Finance Code 001 (88887.236556/2025-00; 88881266909/2026-01).

## Data availability statement

Data are available upon reasonable request. All data relevant to the study are included in the article or uploaded as supplementary information.

## Declaration of competing interest

The authors declare that they have no known competing financial interests or personal relationships that could have appeared to influence the work reported in this paper.
