# Supplementary material for: Is there agreement across diagnostic instruments in the identification of the impairment in intrinsic capacity later in life? A cross-sectional study with community-dwelling older adults
Source: J Nutr Health Aging. 2026 Apr 1;30(5):100829. doi: 10.1016/j.jnha.2026.100829 (PMC13087771; doi:10.1016/j.jnha.2026.100829)
Supplement: Supplementary file 1 [file mmc1.docx]

| **Table S1.** Instruments and scoring criteria for assessment of each IC domain. | |
| --- | --- |
| **Instruments / Description** | **Cutoff point** |
| **COGNITIVE** | |
| **Mini-Mental State Examination (MMSE):** Assesses cognitive function through five domains: spatial-temporal orientation, short-term memory, attention and calculation, language, and spatial construction. The version adapted for the Brazilian population was used [15]. | Education-adjusted[16]  (0) 0-13 [Illiterate]; 0-18 [education<8]; 0-26 [education≥8]: Cognitive decline  (1) 14-30 [Illiterate]; 19-30 [education < 8]; 27-30 [education ≥8]: Normal cognition. |
|  | Raw score of 26 points[7, 17]  (0) ≤ 26: Cognitive decline  (1) >27: Normal cognition |
| **Cognitive battery:** Questions on orientation in time, space, and recall [18]. | (0) Yes: Cognitive decline  (1) No: Normal cognition |
| **LOCOMOTION** | |
| **Short Physical Performance Battery (SPPB):** Assesses timed performance on standing balance, walking speed, and ability to rise from a chair [19]. | (0) 0-9: Limited mobility  (1) 10-12: Normal mobility |
| **Gait speed test:** Participants walked a four-meter distance at a usual, comfortable pace. Timing started when one foot crossed the starting line and ended when one foot crossed the finish line. GS (m/s) was calculated by dividing distance (m) by time (s) [20, 21]. | (0) ≤0.8 m/s: Limited mobility  (1) >0.8 m/s: Normal mobility |
| **Chair stand test:** Participants were asked to stand up five times in a row as quickly as possible from a chair without stopping, keeping arms folded across the chest. Time (in seconds) or inability to perform the test were used for the present analyses [22]. | (0) >15 s or no inability to perform the test: Limited mobility  (1) ≤ 14 s: Normal mobility |
| **Timed Up and Go (TUG) test:** Records the time an individual takes to stand up from a chair, walk three meters, turn around an obstacle, return, and sit down again. Longer times indicate poorer functional performance [23]. | (0) ≥20 s: Limited mobility  (1) <19 s: Normal mobility |
| **VITALITY** | |
| **Mini Nutritional Assessment (MNA):** Nutritional screening tool that determines whether an individual is malnourished or at risk of malnutrition[34]; includes anthropometric assessment, mobility, dietary intake, and health condition[24]. | (0) 0-23.5: Risk of malnutrition/ malnutrition  (1) 24-30: Normal nutritional status |
| **Handgrip Strength (HGS):** Muscle strength was assessed using the Jamar® dynamometer. Participants were instructed to remain seated with the hand at a neutral angle, elbow flexed (90º), and shoulder at a neutral angle; isometric contraction of the dominant hand was requested 3 times, and the average was used for the analyses [25, 26]. | (0) <27 kg (men); <16 kg (women): Low muscle strength  (1) ≥27kg (men); ≥16 kg (women): preserved muscle strength |
| **Body Mass Index (BMI):** Calculated by dividing weight in kilograms by height in meters squared. BMI cutoffs were based on specific cutoff points for older adults in Brazil [27, 28]. | (0) <22 kg/m²: Underweight  (1) ≥22 kg/m²: Eutrophic / Overweight |
| **Self-report:** Self-reported unintentional weight loss (item of MNA) | (0) Unintentional weight loss  (1) No unintentional weight loss |
| **PSYCHOLOGICAL** | |
| **Geriatric Depression Scale (GDS-15):** Quick, accurate tool for detecting depression in older people. Total score range: 0–15 [29, 30]. | (0) 6-15: Depressive symptoms  (1) 0-5: Normal psychological status |
| **Center for Epidemiological Studies for Depression (CES-D)**: 20-item measure that assesses symptoms of depression, with items phrased as self-statements. Respondents rate how frequently each item applied to them over the previous week. Ratings are based on a 4-point Likert scale ranging from 0 (rarely or none of the time [less than 1 day]) to 3 (most or all of the time [5–7 days])[31]. | (0) ≥11: Depressive symptoms  (1) <10: Normal psychological status |
| **Self-report:** Self-reported clinical diagnosis of mental health problem and/or use of antidepressant or anxiolytic medications. | (0) Clinical diagnosis of mental health problem  (1) Without clinical diagnosis of mental health problem |
| **SENSORY** | |
| ***Hearing acuity*** | |
| **Whisper voice test:** Assesses ability to identify or hear whispered words at a distance. Four unrelated words are whispered, and the participant should repeat each word[2]. | (0) 0-2 words: Hearing loss  (1) 3-4 words: Normal hearing capacity |
| **Self-report:** Self-reported hearing impairment. Hearing capacity was considered intact when the participant did not report ‘hearing problems or deafness’ that interfered with their activities, and the interviewer did not identify them as profoundly deaf. | (0) Self-report of hearing problems or deafness  (1) Self-report of normal hearing capacity |
| ***Visual acuity*** |  |
| **Tumbling E chart:** Snellen chart (Optometric scale - Distance = 5.00 m; visual angle = 7; 300 LUX) measures ability to identify or distinguish a letter at a given distance. The Snellen chart contains rows of the letter "E" in various forms of rotation, and the patient is asked to state where the E is pointing, "up, down, left, or right"[32, 33]. | (0) 20/30: Vision impairment  (1) 20/20: Normal visual capacity |
| **Self-report:** Self-reported visual impairment. Vision capacity was considered intact when the participant did not report ‘eyesight problems’ that interfered with their activities and was not identified by the interviewer as functionally blind. | (0) Self-report of eyesight problems  (1) Self-report of normal visual capacity |
| BMI: Body Mass Index; GDS-15: Geriatric Depression Scale; HGS: Handgrip Strength; IC: Intrinsic Capacity; kg: Kilogram; m: Meters; MNA: Mini Nutritional Assessment; MMSE: Mini-Mental State Examination; s: Seconds; SPPB: Short Physical Performance Battery; TUG: Time Up and Go test. | |
