# Supplementary material for: Is there agreement across diagnostic instruments in the identification of the impairment in intrinsic capacity later in life? A cross-sectional study with community-dwelling older adults
Source: J Nutr Health Aging. 2026 Apr 1;30(5):100829. doi: 10.1016/j.jnha.2026.100829 (PMC13087771; doi:10.1016/j.jnha.2026.100829)
Supplement: Supplementary file 2 [file mmc2.docx]

| **Supplementary Table S2.** Pairwise comparisons of Kappa analysis test results between groups. | | | | | | | | | | |
| --- | --- | --- | --- | --- | --- | --- | --- | --- | --- | --- |
| **COGNITIVE** | | | | | | | | **Kappa coefficient** | **Agreement** | **p-value** |
|  | | |  | **MMSE (raw score)** | | | | 0.344 | 62.9% | <0.001 |
|  | | |  | Cognitive decline | | Normal decline | Total |  |  |  |
| **MMSE (education-adjusted** | | | Cognitive decline | 73 (100.0) | | 76 (57.6) | 149 (72.7) |  |  |  |
|  |  |  | Normal decline | 0 (0.0) | | 56 (42.4) | 56 (27.3 |  |  |  |
|  |  |  | Total | 73 (35.6) | | 132 (64.4) | 205 (100) |  |  |  |
|  | | |  | **Cognitive battery** | | | | 0.019 | 36.1% | 0.587 |
|  | | |  | Cognitive decline | | Normal decline | Total |  |  |  |
| **MMSE (education-adjusted)** | | | Cognitive decline | 26 (76.5) | | 123 (71.9) | 149 (72.7) |  |  |  |
|  |  |  | Normal decline | 8 (23.5) | | 48 (28.1) | 56 (27.3) |  |  |  |
|  |  |  | Total | 34 (16.6) | | 171 (83.4) | 205 (100) |  |  |  |
|  | | |  | **Cognitive battery** | | | | 0.094 | 63.4% | 0.127 |
|  | | |  | Cognitive decline | | Normal decline | Total |  |  |  |
| **MMSE (raw score)** | | | Cognitive decline | 16 (47.1) | | 57 (33.3) | 73 (35.6) |  |  |  |
|  |  |  | Normal decline | 18 (52.9) | | 114 (66.7) | 132 (64.4) |  |  |  |
|  |  |  | Total | 34 (16.6) | | 171 (83.4) | 205 (100) |  |  |  |
| **LOCOMOTION** | | | | | | |  | **Kappa coefficient** | **Agreement** | **p-value** |
|  | | |  | | **TUG** | | | 0.683 | 86.3% | <0.001 |
|  | | |  | | Normal mobility | Limited mobility | Total |  |  |  |
| **SPPB** | | | Normal mobility | | 127 (87.0) | 9 (15.3) | 136 (66.3) |  |  |  |
|  |  |  | Limited mobility | | 19 (13.0) | 50 (84.7) | 69 (33.7) |  |  |  |
|  |  |  | Total | | 146 (71.2) | 59 (28.8) | 205 (100) |  |  |  |
|  | | |  | | **Gait speed test** | | | 0.612 | 83.4% | <0.001 |
|  | | |  | | Normal mobility | Limited mobility | Total |  |  |  |
| **SPPB** | | | Normal mobility | | 125 (84.5) | 11 (19.3) | 136 (66.3) |  |  |  |
|  |  |  | Limited mobility | | 23 (15.5) | 46 (80.7) | 69 (33.7) |  |  |  |
|  |  |  | Total | | 148 (72.2) | 57 (27.8) | 205 (100) |  |  |  |
|  | | |  | | **Chair stand test** | | | 0.711 | 87.8% | <0.001 |
|  | | |  | | Normal mobility | Limited mobility | Total |  |  |  |
| **SPPB** | | | Normal mobility | | 131 (86.8) | 5 (9.3) | 136 (66.3) |  |  |  |
|  |  |  | Limited mobility | | 20 (13.2) | 49 (90.7) | 69 (33.7) |  |  |  |
|  |  |  | Total | | 151 (73.7) | 54 (26.3) | 205 (100) |  |  |  |
|  | | |  | | **Gait speed test** | | | 0.760 | 90.2% | <0.001 |
|  | | |  | | Normal mobility | Limited mobility | Total |  |  |  |
| **TUG** | | | Normal mobility | | 137 (92.6) | 9 (15.8) | 146 (71,2) |  |  |  |
|  | | | Limited mobility | | 11 (7.4) | 48 (84.2) | 59 (28.8) |  |  |  |
|  | | | Total | | 148 (72.2) | 57 (27.8) | 205 (100) |  |  |  |
|  | | |  | | **Chair stand test** | | | 0.597 | 83.9% | <0.001 |
|  | | |  | | Normal mobility | Limited mobility | Total |  |  |  |
| **TUG** | | | Normal mobility | | 132 (87.4) | 14 (25.9) | 146 (71.2) |  |  |  |
|  | | | Limited mobility | | 19 (12.6) | 40 (74.1) | 59 (28.8) |  |  |  |
|  | | | Total | | 151 (7372) | 57 (27.8) | 205 (100) |  |  |  |
|  | | |  | | **Chair stand test** | | | 0.494 | 80.0% | <0.001 |
| **Gait speed test** | | |  | | Normal mobility | Limited mobility | Total |  |  |  |
|  |  |  | Normal mobility | | 129 (85.4) | 19 (35.2) | 148 (72.2) |  |  |  |
|  |  |  | Limited mobility | | 22 (14.6) | 35 (64.8) | 57 (27.8) |  |  |  |
|  |  |  | Total | | 151 (73.7) | 54 (26.3) | 205 (100) |  |  |  |
| **VITALITY** | | | | | | |  | **Kappa coefficient** | **Agreement** | **p-value** |
|  | | |  | | **HGS** | |  | 0.176 | 71.7% | 0.011 |
|  | | |  | | Normal nutrition | Undernutrition | Total |  |  |  |
| **MNA** | | | Normal nutrition | | 131 (79.9) | 25 (61.0) | 156 (76.1) |  |  |  |
|  |  |  | Undernutrition | | 33 (20.1) | 16 (39.0) | 49 (23.9) |  |  |  |
|  |  |  | Total | | 164 (80.0) | 41 (20.0) | 205 (100) |  |  |  |
|  | | |  | | **BMI** | |  | 0.223 | 77.1% | <0.001 |
|  | | |  | | Normal nutrition | Undernutrition | Total |  |  |  |
| **MNA** | | | Normal nutrition | | 146 (79.8) | 10 (45.5) | 156 (76.1) |  |  |  |
|  |  |  | Undernutrition | | 37 (20.2) | 12 (54.5) | 49 (23.9) |  |  |  |
|  |  |  | Total | | 183 (89.3) | 22 (10.7) | 205 (100) |  |  |  |
|  | | |  | | **Self-report** | |  | 0.293 | 74.6% | <0.001 |
|  | | |  | | Normal nutrition | Undernutrition | Total |  |  |  |
| **MNA** | | | Normal nutrition | | 131 (82.9) | 25 (53.2) | 156 (76.1) |  |  |  |
|  |  |  | Undernutrition | | 27 (17.1) | 22 (46.8) | 49 (23.9) |  |  |  |
|  |  |  | Total | | 158 (77.1) | 47 (22.9) | 205 (100) |  |  |  |
|  | | |  | | **BMI** | | | -0.015 | 79.0% | 0.821 |
|  | | |  | | Normal nutrition | Undernutrition | Total |  |  |  |
| **HGS** | | | Normal nutrition | | 146 (79.8) | 18 (81.8) | 164 (80.0) |  |  |  |
|  |  |  | Undernutrition | | 37 (20.2) | 4 (18.2) | 41 (20.0) |  |  |  |
|  |  |  | Total | | 183 (89.3) | 22 (10.7) | 205 (100) |  |  |  |
|  | | |  | | **BMI** | | | 0.134 | 75.1% | 0.034 |
|  | | |  | | Normal nutrition | Undernutrition | Total |  |  |  |
| **Self-report** | | | Normal nutrition | | 145 (79.2) | 13 (59.1) | 158 (77.1) |  |  |  |
|  |  |  | Undernutrition | | 38 (20.8) | 9 (40.9) | 47 (22.9) |  |  |  |
|  |  |  | Total | | 158 (77.1) | 22 (10.7) | 205 (100) |  |  |  |
|  | | |  | | **Self-report** | | | 0.075 | 68.8% | 0.280 |
|  | | |  | | Normal nutrition | Undernutrition | Total |  |  |  |
| **HGS** | | | Normal nutrition | | 146 (79.8) | 18 (81.4) | 164 (80.0) |  |  |  |
|  |  |  | Undernutrition | | 37 (20.2) | 4 (18.2) | 41 (20.0) |  |  |  |
|  |  |  | Total | | 183 (89.3) | 22 (10.7) | 205 (100) |  |  |  |
| **PSYCHOLOGICAL** | | | | | | |  | **Kappa coefficient** | **Agreement** | **p-value** |
|  | |  | | | **CES-D** | | | 0.270 | 70.7% | <0.001 |
|  | |  | | | Normal psychological | Depressive symptoms | Total |  |  |  |
| **GDS-15** | | Normal psychological | | | 124 (95.4) | 54 (72.0) | 178 (86.8) |  |  |  |
|  |  | Depressive symptoms | | | 6 (4.6) | 21 (28.0) | 27 (13.2) |  |  |  |
|  |  | Total | | | 130 (63.4) | 75 (36.6) | 205 (100) |  |  |  |
|  | |  | | | **Self-report** | | | 0.081 | 71.7% | 0.218 |
|  | |  | | | Normal psychological | Depressive symptoms | Total |  |  |  |
| **GDS-15** | | Normal psychological | | | 138 (88.5) | 40 (81.6) | 178 (86.8) |  |  |  |
|  |  | Depressive symptoms | | | 18 (11.5) | 9 (18.4) | 27 (13.2) |  |  |  |
|  |  | Total | | | 156 (75.1) | 49 (23.9) | 205 (100) |  |  |  |
|  | |  | | | **Self-report** | | | 0.160 | 63.9% | 0.016 |
|  | |  | | | Normal psychological | Depressive symptoms | Total |  |  |  |
| **CES-D** | | Normal psychological | | | 106 (67.9) | 24 (49.0) | 130 (63.4) |  |  |  |
|  |  | Depressive symptoms | | | 50 (32.1) | 25 (51.0) | 75 (36.6) |  |  |  |
|  |  | Total | | | 156 (75.1) | 49 (23.) | 205 (100) |  |  |  |
| **SENSORY (hearing)** | | | | | | | | **Kappa coefficient** | **Agreement** | **p-value** |
|  | | |  | **Self-report** | | |  | 0.689 | 88.8% | <0.001***** |
|  | | |  | Normal hearing | | Hearing loss | Total |  |  |  |
| **Whisper voice test** | | | Normal hearing | 145 (91.8) | | 10 (21.3) | 155 (75.6) |  |  |  |
|  |  |  | Hearing loss | 13 (8.2) | | 37 (78.7) | 50 (24.4) |  |  |  |
|  | | | Total | 158 (77.1) | | 47 (22.9) | 205 (100) |  |  |  |
| **SENSORY (vision)** | | | | | | | | **Kappa coefficient** | **Agreement** | **p-value** |
|  | | |  | **Self-report** | | | | -0.040 | 35.1% | 0.335 |
|  | | |  | Normal visual | | Vision impairment | Total |  |  |  |
| **Snellen chart** | | | Normal visual | 43 (26.7) | | 15 (34.1) | 58 (28.3) |  |  |  |
|  |  |  | Vision impairment | 118 (73.3) | | 29 (65.9) | 147 (71.7) |  |  |  |
|  |  |  | Total | 161 (78.5) | | 44 (21.7) | 205 (100) |  |  |  |
|  | BMI: Body Mass Index; GDS-15: Geriatric Depression Scale; HGS: Handgrip Strength; IC: Intrinsic Capacity; kg: Kilogram; m: Meters; MNA: Mini Nutritional Assessment; MMSE: Mini-Mental State Examination; s: Seconds; SPPB: Short Physical Performance Battery; TUG: Time Up and Go test. Agreement analysis: K = 0–0.20: no agreement; K = 0.21–0.39: minimal agreement; K = 0.40–0.59: weak agreement; K = 0.60–0.79: moderate agreement; K = 0.80–0.90: strong agreement; K > 0.90: nearly perfect agreement. | | | | | | | | | |
